# Positive Epistasis Drives the Acquisition of Multidrug Resistance

**DOI:** 10.1371/journal.pgen.1000578

**Published:** 2009-07-24

**Authors:** Sandra Trindade, Ana Sousa, Karina Bivar Xavier, Francisco Dionisio, Miguel Godinho Ferreira, Isabel Gordo

**Affiliations:** 1 Instituto Gulbenkian de Ciência, Oeiras, Portugal; 2 Departamento de Biologia Vegetal and Centro de Biologia Ambiental, Faculdade de Ciências, Universidade de Lisboa, Campo Grande, Lisboa, Portugal; 3 Instituto de Tecnologia Química e Biológica, Universidade Nova de Lisboa, Oeiras, Portugal; University of Michigan, United States of America

## Abstract

The evolution of multiple antibiotic resistance is an increasing global problem. Resistance mutations are known to impair fitness, and the evolution of resistance to multiple drugs depends both on their costs individually and on how they interact—epistasis. Information on the level of epistasis between antibiotic resistance mutations is of key importance to understanding epistasis amongst deleterious alleles, a key theoretical question, and to improving public health measures. Here we show that in an antibiotic-free environment the cost of multiple resistance is smaller than expected, a signature of pervasive positive epistasis among alleles that confer resistance to antibiotics. Competition assays reveal that the cost of resistance to a given antibiotic is dependent on the presence of resistance alleles for other antibiotics. Surprisingly we find that a significant fraction of resistant mutations can be beneficial in certain resistant genetic backgrounds, that some double resistances entail no measurable cost, and that some allelic combinations are hotspots for rapid compensation. These results provide additional insight as to why multi-resistant bacteria are so prevalent and reveal an extra layer of complexity on epistatic patterns previously unrecognized, since it is hidden in genome-wide studies of genetic interactions using gene knockouts.

## Author Summary

Understanding the nature of genetic interactions, known as epistasis, is crucial in biology. The strength and type of epistasis is relevant for the evolution of sex, buffering of genetic variation, speciation, and the topography of fitness landscapes. While epistasis between gene deletions has been the recent focus of research, interactions between randomly selected alleles, which are of the greatest evolutionary interest, have not. We have studied the strength and type of epistasis amongst alleles that confer antibiotic resistance and have found that: in an antibiotic-free environment, the cost of multiple resistance is smaller than expected—a signature of pervasive positive epistasis amongst alleles that confer resistance to antibiotics; epistatic interactions are allele specific; a significant fraction of resistant mutations can be beneficial in certain resistant genetic backgrounds; some double resistances entail no measurable cost; and some allelic combinations are hotspots for rapid compensation. Overall, our findings provide added reasoning as to why multi-resistance is so difficult to eradicate. Importantly, our results of allelic-specific epistasis reveal an extra layer of complexity on epistatic patterns previously unrecognized.

## Introduction

Epistasis occurs when the phenotypic effect of a mutation in a locus depends on which mutations are present at other loci. When the phenotype of interest is fitness, the existence of such genetic interactions can constrain the course of evolution. The strength and form of epistasis is relevant for the evolution of sex, buffering of genetic variation, speciation and the topography of fitness landscapes. While epistasis between gene deletions [1],[2] has been the focus of recent research, interactions between randomly selected alleles, which are of the greatest evolutionary interest has not [3]. Since mutations that confer antibiotic resistance are known to affect bacterial fitness, levels of epistasis amongst such mutations may determine how multiple resistance evolves. Such knowledge can be used to understand and predict what type of resistance mutations are likely to be segregating in microbial populations [4]. To understand and predict the evolution of multiple resistance it is of key importance to know how the fitness of sensitive and resistance bacteria is affected in different environments, particularly both in the presence and in the absence of drugs. Recent studies have shown that interactions exist amongst pairs of antibiotics, i.e. resistance to one drug affects the action of another drug [5],[6]. In particular the combination of pairs of drugs has been studied and the combinations have been characterized as additive, synergistic, antagonistic or suppressive. Importantly it has been found that in certain drug combinations (suppressive) one of the antibiotics may render the treatment more effective against its resistant mutant than against the wild type [6]. However, we are lacking data on genetic interactions amongst single nucleotide mutations conferring antibiotic resistance in a drug free environment, i.e. the cost of multiple resistance. With the occurring increase in frequency of multiple resistant bacteria and the public health problems associated with it, knowledge on the type and strength of epistasis is of most importance in understanding the evolution of multiple resistance and, ultimately, the planning of new strategies for human intervention.

One can think that a way to halt the spread of resistance to a given antibiotic is to stop the use of that antibiotic. In the absence of the antibiotic for which resistance has been acquired, antibiotic-resistance mutants have a fitness cost when compared to sensitive bacteria [7],[8]. However, when infection is not resolved, a common strategy is to continue treatment with a different antibiotic to which the infecting bacteria are still susceptible. Unfortunately this strategy has led to rapid increase in multiple-drug resistance and not to the loss of resistance to the first treatment, as it would be desired [9]. This raises the possibility that multi-resistant mutations are not independent. If so, when resistance first develops the following question should be asked: if a pathogenic strain is resistant to antibiotic X, which antibiotic should be administered as a second treatment? Clearly, the strategy will depend not only on knowledge about the fitness costs of single resistance mutations but also on the level of genetic interactions – epistasis - between the alleles that underlie those phenotypes. When epistasis exists, it can be positive (antagonistic or alleviating) or negative (synergistic or aggravating). If strong negative epistasis amongst drug resistance alleles is found, then the cost of multiple resistances is high and one can expect multi-drug resistant microbes to be counter selected and disappear very rapidly in the absence of either drug. On the contrary, a much more worrying scenario is the existence of positive epistasis, for it implies that the expected time for elimination of multiple resistance, even if antibiotic pressure is inexistent, will be much longer and multiple resistant bacteria are expected to accumulate in the population.

Here we quantify the degree of genetic interactions on cellular fitness in an antibiotic free environment for point mutations which confer resistance to commonly used antibiotics of three different classes. We have focused on resistances to: (i) the quinolone nalidixic acid, which inhibits DNA replication by binding to DNA gyrase; (ii) rifampicin, which belongs to the rifamycins class of antibiotics that bind to the β-subunit of RNA polymerase thereby inhibiting transcription; and (iii) streptomycin, an aminoglycoside that binds to the ribosome and inhibits elongation of protein synthesis [10]. We find that epistasis is allele specific and that the vast majority of allelic combinations exhibit positive epistasis. The costs of double resistance are therefore smaller than what one would expect if they were independent. Interestingly we found several cases of sign epistasis, which implies that mutations conferring resistance to a new antibiotic are compensatory, i.e. alleviate the cost of resistance present on another locus.

## Results

### Single-resistant mutants' fitness costs

To study the degree of epistatic interactions amongst alleles that confer antibiotic resistance we started by selecting a series of *Escherichia coli* spontaneous mutants resistant to commonly used antibiotics of 3 different classes: nalidixic acid, rifampicin, and streptomycin (Methods). From a panel of 120 sequenced clones that carry a single nucleotide change, we obtained 19 different classes of clones (Table S1) with spontaneous mutations in *gyrA*, *rpoB* and *rpsL*, which are the correspondent common target genes of resistance to nalidixic acid, rifampicin, and streptomycin, respectively. Resistant bacteria with mutations in the same amino acids as those collected here are segregating in microbial populations [4],[11]. We should notice that our procedure for isolating clones carrying antibiotic resistance requires that viable colonies of resistant bacteria can be formed and detected. Any mutations that can arise and cause very high fitness costs cannot therefore be accounted for in this study. Given that highly deleterious mutations are unlikely to segregate in natural populations [4], genetic interactions amongst such mutations are likely to be of less clinical importance, unless these can be very easily compensated for.

**Figure 1:**
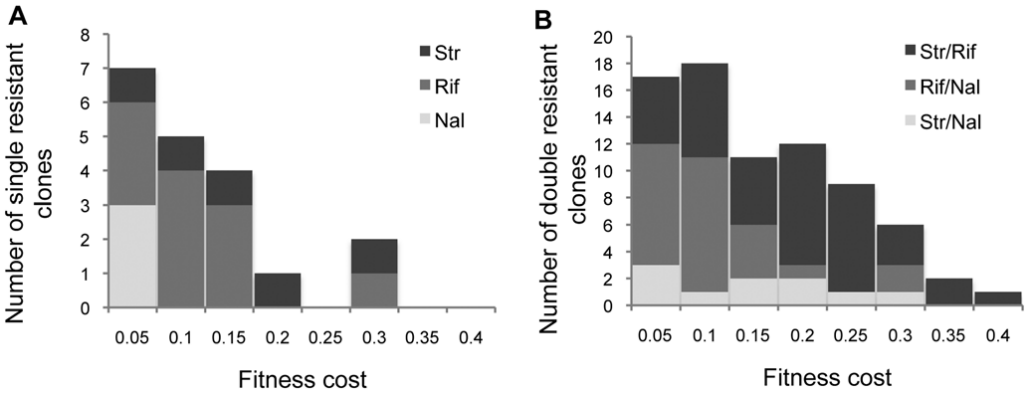
Distribution of fitness costs of single and double mutants indicative of positive epistasis. (A) Distribution of fitness costs of clones carrying single point mutations that confer resistance to streptomycin (black), rifampicin (dark grey) and nalidixic acid (light grey). (B) Distribution of fitness costs of clones carrying double resistance to streptomycin/rifampicin (black), rifampicin/nalidixic acid (dark grey) and streptomycin/nalidixic acid (light grey). The mean fitness cost of double resistants is less than twice the mean cost of single resistance mutations.

We determined the cost of resistance of each of the clones in the absence of antibiotics. This was performed by measuring relative competitive fitness of the resistant strains against a marked sensitive strain through a competition assay in liquid LB medium without antibiotics [12] (Methods). Figure 1A shows the distribution of fitness costs of each of the mutants. The genotype of each mutation as well as its fitness costs are provided in Table S1. On average the cost of antibiotic resistance was 9%. A Kolmogorov-Smirnov test does not reject the exponential distribution (P = 0.85), a commonly used model to describe the distribution of deleterious mutations in several organisms [13]. Mutations that confer resistance to nalidixic acid had a lower average fitness cost (3%) than streptomycin or rifampicin (13% and 9%, respectively). Similar levels of fitness cost were found previously in resistant mutants of *Mycobacterium tuberculosis* [4],[14] and *E. coli* [15],[16].

### Epistasis between deleterious mutations that confer antibiotic resistance

To study the fitness cost of double resistance and measure epistasis we constructed by P1 transduction all possible pairwise combinations (103) between different resistance alleles. These correspond to 5×11 streptomycin/rifampin, 5×3 streptomycin/nalidixic acid and 11×3 rifampin/nalidixic acid possible combinations. We measured the fitness of the obtained double resistant by competition assays, determined its cost and compared it with the cost that would be predicted if there was no epistasis. Figure 1B shows that the average cost of double resistance is less than twice the average cost of a single resistance (compare with Figure 1A), indicating positive epistasis. Fitness of double resistance was used to calculate pairwise epistasis. Pairwise epistasis, *ε*, between locus A and B can be measured as follows [17]. If *W_AB_* is the fitness of the wildtype, *W_Ab_*, *W_aB_* are the fitnesses of each of the single mutants and *W_ab_* that of the double mutant then:
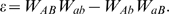
When the value of the costs is small then this measure becomes very similar to the difference between the cost of double resistance and the sum of the costs of each resistance.

**Figure 2:**
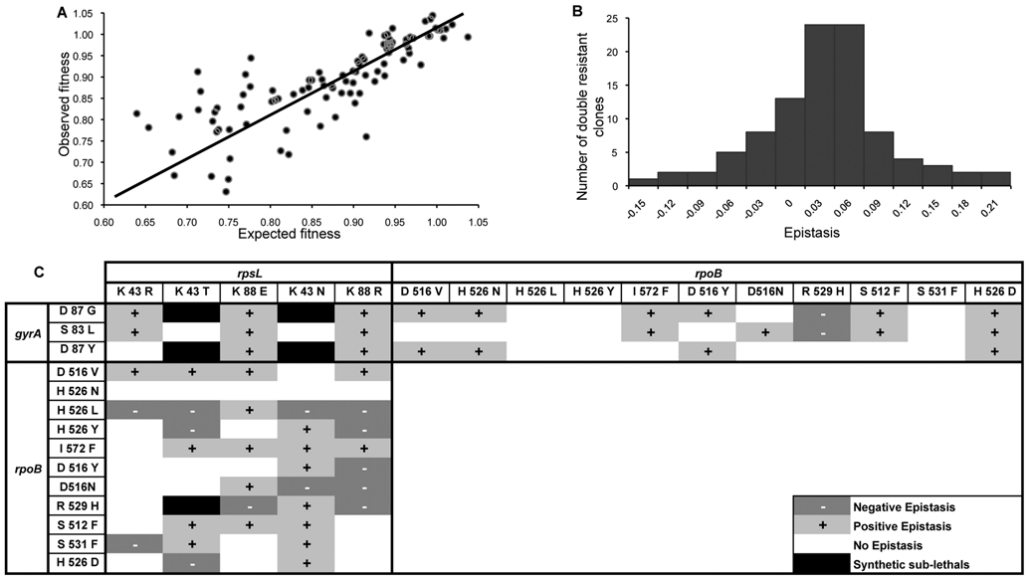
Evidence for positive epistasis. (A) Relation between the observed fitness of the double resistance genotypes and the expected fitness under the assumption of no epistasis. (B) Distribution of the epistasis level *ε*, whose median is 0.025 with bootstrap confidence interval [0.016, 0.032], showing positive epistasis. (C) Allelic dependence of epistasis between the *rpsL*, *rpoB* and *gyrA* (positive epistasis in light grey, negative dark grey and not significant in white. Black indicates combinations of alleles for which there was a low efficiency of transduction - synthetic sub-lethals).

Strikingly, the majority of mutations show positive epistasis. 68% of the points are above the line in Figure 2A and from the clones that show significant epistasis 15% show negative epistasis and 42% show positive epistasis. The later ones correspond to clones where the cost is less than the sum of the costs of each resistance (see also Figure 2C for the specific combinations of alleles showing significant positive epistasis).

It has been suggested from theoretical modeling of RNA secondary structures and from studies in digital organisms – computer programs that mutate and evolve- [18] that epistasis and the fitness effect of mutations may be correlated, such that mutations with larger effects are more epistatic. In our data we find significant correlations between the strength of epistasis (deviation from zero in absolute value) and the costs of the mutations (Pearson's correlation r = 0.61, P<0.001), which support this theoretical prediction. We also find a marginally significant correlation between the value of epistasis, *ε*, and the cost of the mutations (Pearson's correlation r = 0.19, P = 0.06).

Figure 2B shows the distribution of the *ε* values, the median is significantly positive (median = 0.025, Bootstrap 95% CI [0.016; 0.032]), and its value corresponds to about 1/3 of the average cost of each single mutation. From the allelic combinations for which there is significant epistasis (53%), only 27% give rise to negative epistasis, whereas 73% of these combinations result in positive epistasis. Epistatic interactions were observed more frequently between mutations in *gyrA* and in *rpsL*, with high frequency of positive epistasis but also extreme cases of negative epistasis (synthetic sub-lethal, Figure 2C). Combination of *gyrA* and *rpoB* mutations produced the lowest frequency of negative epistasis (6%), which suggests that the sequential prescription of antibiotics leading to these resistances may easily result in multi-resistance development.

Focusing on the mutations we notice that the interactions are not gene but allele specific. For example *R529H* and *H526L* mutations in *rpoB* notably showed very high negative epistasis frequency, whereas four other mutations (*D516V*, *H526N*, *I572F* and *S512F*) in the same gene showed no negative epistasis regardless of the combination, and *D516V* and *I572F* mutations revealed high frequency of positive epistasis. The latter are potential candidates to segregate in natural populations. The *rpoB H526D* mutation is a specific example where its epistasis is highly dependent on the particular allele of the second mutation: *rpoB H526D* interacts positively with *rpsL K43N*, but negatively with *rpsL K43T*. Interestingly, the *rpoB H526D* mutation has been found in multi-drug resistant *M. tuberculosis* strains and also in this organism its cost was shown to depend on the genetic background [4]. This suggests two important predictions: that we should find resistance alleles with strong positive interaction segregating at higher frequencies (such as *rpoB H526D*/*rpsL K43N*) in natural populations and that these genetic interactions should also apply to microorganisms other than *E. coli*.

### Sign epistasis in antibiotic resistance mutations

**Figure 3:**
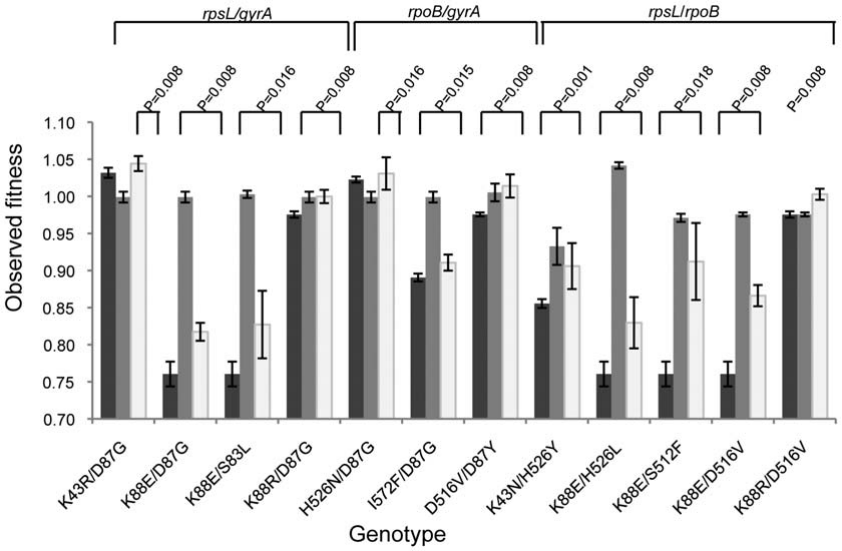
Evidence for sign epistasis amongst alleles conferring resistance. Sign epistasis occurs when the fitness of the double mutant (white bar) is greater than the fitness of at least one single mutant (dark grey and light grey bars). The genotypes of the double mutants where we found sign epistasis are indicated below the bars and the P values of the Wilcoxon test are indicated above the bars.

Recently it has been shown that an important type of epistasis, which is known as sign epistasis [19], may constrain the evolution of resistance to high penicillin concentrations [20]. Sign epistasis happens when the sign of the fitness effect of a mutation (deleterious or beneficial) is itself epistatic, i.e. sign epistasis exists when a mutation is deleterious on some genetic backgrounds but beneficial on others. This form of epistasis, if common, may give rise to multiple peaks on the fitness landscape. In the context of antibiotic resistance, the implication is simple: if sign epistasis is pervasive then it will be much more difficult to move from a scenario of multiple drug resistance to a scenario where all the bacteria are sensitive, even if antibiotic selection pressure is stopped. Experimental evolution studies have shown that, within hundreds of generations, mutations which compensate for the cost of antibiotic resistance (compensatory) are more likely to occur [16],[21],[22] than revertants [14]. This suggests that sign epistasis might have an important role for the evolution of antibiotic resistance. Within the allelic combinations studied, 12% of our clones showed unexpected sign epistasis between drug resistance alleles. This corresponds to double mutants that have a fitness bigger than the fitness of at least one of the single mutants (Figure 3), and here it means that the mutation conferring resistance to a new antibiotic is beneficial (compensatory) when in a genetic background that contains a mutation conferring resistance to a different antibiotic. This is the worst possible scenario for the host and the best possible for the microbe. Given that a particular mutation was just selected by application of an antibiotic, evolution by natural selection makes it likely that the fixation of a mutation conferring resistance to another antibiotic will occur, even if selective pressure is not applied. Specifically, we find that the same mutation conferring streptomycin resistance (*rpsL K88E*, Figure 3) can be compensated by different mutations that confer rifampicin resistance showing that rifampicin treatment should be avoided in patients infected with *rpsL K88E* streptomycin resistant mutants. Given these results, knowledge of both the clinical history of patient antibiotic use as well as the specific genotypes associated with a given resistance is recommended for predicting the optimal clinical outcomes.

Another worrying class of clones that we found corresponds to combination of double resistant mutations that entail no significant cost. These are 6 out of the 103: *rpsL K43R*/*gyrA D87G*, *rpsL* K43R/*gyrA D87Y*, *rpoB H526N*/*gyrA D87G*, *rpoB H526N*/*gyrA D87Y*, *rpoB D516V*/*gyrA D87Y*, *rpsL K43R*/*rpoB H526N*. These mutants have no disadvantage against the wild-type. Although this constitutes a small percentage, it is nevertheless of extreme importance since for these double mutants little, if any, compensation is required for restoring the competitive ability of the wild-type. Given the known association between the cost of resistance mutations measured in the laboratory and the frequency at which they are found in clinical settings has been demonstrated, at least in *M. tuberculosis* [4], we predict that those combinations of double mutations are the ones which are more likely to be found. Future studies are planned to test this prediction, although it should be noted that the target for resistance may vary between species and environmental conditions [23]. In the opposite extreme we found five clones for which the transduction efficiency was very low (combinations shown in black, Figure 2C) which might correspond to combinations of mutations that must entail high fitness cost.

### Level and pattern of epistasis in spontaneous double-resistant clones

**Figure 4:**
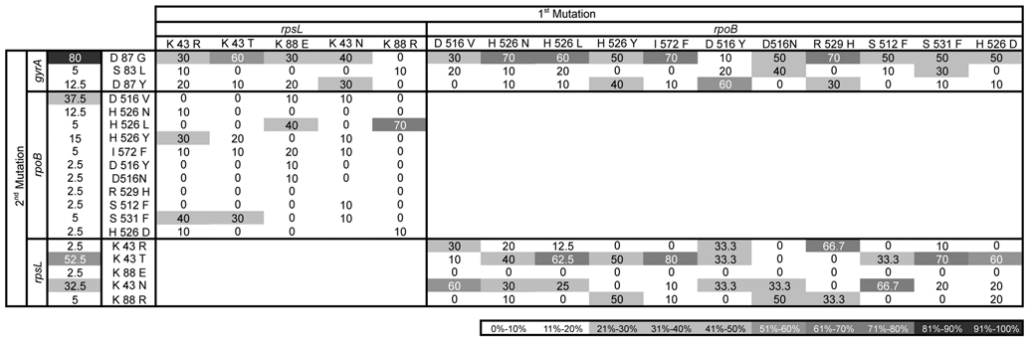
Mutational spectrum and frequency of spontaneous double resistance mutations. Given the genetic background of the first mutation (column) the frequency of the second mutation (raw) appears as percentage in grey scale gradient. For a given background the percentage of mutations in the second locus adds up to 100%, in most cases, except when the second mutation occurred in a different gene. The percentage of the occurrence of each mutation in a wild-type background is also shown in the third column.

To make predictions about which resistance alleles are likely to be segregating it is important to study the frequency at which they spontaneously arise. To query which of the P1 transducted mutants are likely to naturally occur and if these also show pervasive epistasis we collected hundreds of spontaneous double resistance mutants. From 289 clones that were sequenced, we obtained 76 different genotypes, whose frequencies are given in Figure 4. We performed a χ^2^-square test to investigate the effects of genetic background on the spectrum of mutations that spontaneously arise. We observe that there are significant differences between the types of new resistance mutations that appear in certain resistant backgrounds and the wild-type sensitive background (Table S2).

We measured the fitness of each of the different spontaneous double resistant clones and compared it with the fitness in the corresponding clones constructed by P1 transduction. 67 out of 72 spontaneous clones were not different from the P1 constructed mutants (Figure S2), but surprisingly, five double resistant clones (*rpoB S531F*/*gyrA D87Y*; *rpsL K43T*/*rpoB H526Y*; *rpsL K88R*/*rpoB H526D*; *rpsL K43R*/*rpoB S531F*; *rpoB I572F*/*rpsL K88R*) had a significantly higher fitness (Wilcoxon test, P<0.01). These five spontaneous mutants have a higher fitness because they must have acquired an extra mutation during their isolation which is compensatory. To show that this is in fact the case we measured the fitness of independent clones carrying the same resistance mutations. For three of these haplotypes (*rpsL K43T*/*rpoB H526Y*, *rpsL K88R*/*rpoB H526D*, *rpsL K43R*/*rpoB S531F*) we measured the fitness of spontaneous clones which were obtained applying the reversed antibiotic selection procedure. (i.e. the clones were now isolated by selecting first for rifampicin and secondly for streptomycin). A Wilcoxon-test revealed that the fitness values of these new independent clones were not different from the corresponding P1 clones, showing that the original spontaneous clones carry a compensatory mutation. Given the reduced number of generations in the procedure of generating spontaneous double resistant mutants, observing a compensatory mutation is only probable if such mutation has a very strong effect (s_c_). This is because only with a large s_c_ it will not be stochastically lost (probability of fixation ∼2s_c_), and can fix in such short time (time to fixation ∼1/s_c_) [24]. Indeed for the five mutants mentioned above, the estimated fitness effect s_c_ is very large, on average 0.09 (with the corresponding effects of each compensatory mutation 0.07; 0.13; 0.11; 0.07; 0.06). Adaptive mutations of such strong effect emerging and fixing so rapidly in bacterial populations under such small effective population size, is surprising given previous estimates of effects of beneficial mutations (on average 0.01) [25]. A strong compensatory mutation must also have occurred in the spontaneous clones carrying *rpsL K43T*/*gyrA D87G*, *rpsL K43N*/*gyrA D87G*, *rpsL K43T*/*gyrA D87Y* and *rpsL K43N*/*gyrA D87Y* mutations, which were determined as synthetic sub-lethals by P1 transduction.

We calculated the *ε* values for the 67 spontaneous clones where we do not have evidences for extra compensatory mutations and obtained the same trend as with the double mutants constructed by P1 transduction (58% showed significant epistasis from which 74% showed positive epistasis and 7% that had no significant cost (Figure S3)). These results indicate that positive epistasis is also pervasive in spontaneous mutants supporting the relevance of our results. Additionally, there is evidence from epidemiological studies that in *M. tuberculosis* environmental resistant isolates more than 96% of the strains resistant to rifampicin have at least one mutation in *rpoB*, 52 to 59% of the streptomycin resistant strains have mutations in *rpsL* and 74 to 94% of the strains resistant to ofloxacin or levofloxacin (quinolones like nalidixic acid used here) have mutations in *gyrA* [26]. Also, the same type of mutations have been isolated in *E. coli* [27] and in *Salmonella enterica* where 42% of the isolates showed substitutions in *gyrA* at position S83 and 35% at position D87 [28], that we show here to exhibit epistasis. Thus we expect the traits observed to be relevant for the evolution of multi-drug resistance acquired during treatment of infectious agents.

## Discussion

Given the importance of epistasis in a variety of biological features such as sex and recombination, buffering of genetic variation, speciation, and the evolution of genetic architecture [29], recent studies have focused on measuring genome-wide levels of epistasis. However, typically only deletions or knockout mutations were studied [1],[2],[30],[31]. Here, albeit focusing in a small number of genes, we measured epistasis on fitness at the scale of the allele given that these may be the most common type of mutations segregating in natural populations and because it also allows us to study mutations in essential genes. Our results not only show the significance of these interactions for the evolution of antibiotic resistance but also reveal an extra layer of complexity on epistatic patterns previously unpredicted, since it is hidden in genome-wide studies of genetic interactions using gene knockouts.

The data obtained here revealed an average level of positive epistasis, which differs strongly from the results of the degree of epistasis among slightly deleterious mutations caused by random transposon insertions in *E. coli*, where on average no epistasis was found [30]. Positive epistasis has also been found in HIV-1 isolates [32]. Since the data in that study was obtained by sampling mutants from natural populations, comparisons between the results obtained and those found here should be taken carefully. We note nevertheless that some bias towards positive epistasis that may be present in the HIV study is not present in our study, since we constructed all possible pairwise combinations of double mutants. It remains to be explored whether the type of mutations (single nucleotide changes, deletions or tranpositions) can affect the pattern of genetic interaction which can be observed. We predict that it can since we show that the type of interactions is not gene but allele specific. Positive epistasis was also detected when studying interactions between rifampin and streptomycin resistant mutants in *Pseudomonas aeruginosa* [33]. The pattern in *E. coli* presented here and the results found in *P. aeruginosa* (even though having a different genetic basis) [33] indicate that the presence of positive epistasis amongst antibiotic resistance mutations is not species specific. Furthermore epistatic interactions involving fluoroquinolone resistance mutations in *gyrA* have also been found in *Streptococcus pneumoniae* [34].

Although we have studied different classes of antibiotics that affect different cell targets, our finding of pervasive epistasis can be reflecting the fact that these target genes are part of the fundamental flow from DNA to RNA to protein, and thus can be considered to be working in the same pathway. Because these affect highly conserved cell processes they should be relevant in many different organisms. An interaction between some of these genes has been described for specific traits, namely propagation of bacteriophage T7 (interaction between *rpsL* and *rpoB*) and mitomycin C resistance in *E. coli* (between *rpoB* and *gyrA*) [35],[36].

Our data, both of spontaneous and P1 transducted double resistant clones, also indicates the presence of sign epistasis in the cost of multi-drug resistance involving rifampin, streptomycin and nalidixic acid, that is, a small fraction of double resistant clones showed a higher fitness than at least one of the corresponding single resistant mutants. Sign epistasis implies that the fixation of one mutation (for example by strong selection pressure of a given antibiotic) may alter the adaptive path in both number and type of subsequent beneficial mutations. Sign epistasis was also previously found in the context of resistance to the antibiotic cefotaxime [20]. In this system, of the 120 possible mutational paths from the low resistance to high resistance, only 18 can actually occur due to the occurrence of sign epistasis. Although there are not many examples in the literature [19], this one clearly shows the power of sign epistasis in constraining protein adaptation.

Another interesting example in the context of this work is bacterial adaptation to the cost of resistance through the acquisition of new compensatory mutations. In such an adaptive process it was observed that adaptive mutations which reduce the cost of resistance to streptomycin in *E. coli* [37] and Salmonella [12] are deleterious in the streptomycin-sensitive background and therefore constitute an example of sign epistasis.

In this work we have determined the fitness effect of mutations in the absence of antibiotics. Future studies should focus on epistatic interactions when bacteria grow in the presence of antibiotics, a condition already shown to be relevant to the evolution of resistance [38],[39].

Our results highlight the importance of determining the costs of single and multiple resistances and, accordingly ordering allele combinations by the degree of epistasis they exhibit. Given that at the present time, infections are likely to be caused by microbes that carry resistance to at least one drug, the strategy expected to give the best outcome is one in which the next drug is the one leading simultaneously to the resistant mutant with the biggest cost and strongest negative epistasis. Another approach to prevent the evolution of multidrug resistance would be to use drug combinations (at certain concentrations) that select for sensitive bacteria. This is a plausible scenario since it has been shown that when competing in the presence of the two drugs, sensitive bacteria outgrow resistant [6]. Our finding of pervasive positive epistasis suggests one possible explanation for the difficulty of eradicating multi-drug resistance in organisms like *M. tuberculosis*, for which current treatments involve combinations of the same drugs as studied here.

## Methods

### Bacterial strains and growth conditions

The strains used were *Escherichia coli* K12 MG1655 and *Escherichia coli* K12 MG1655 Δara. All clones with antibiotic resistant mutations were derived from the ancestral strain *Escherichia coli* K12 MG1655. *Escherichia coli* K12 MG1655 Δara, was used as reference for the competition fitness assay. The two strains are distinguishable by phenotypic difference due to a deletion in the arabinose operon: ara^+^ and Δara give rise to white and red colonies, respectively, in tetrazolium arabinose (TA) indicator agar [12]. The clones were grown at 37°C on plates containing Luria-Bertani (LB) supplemented with agar and the respective antibiotics. The antibiotic concentrations were 100 µg/ml for rifampicin, 40 µg/ml for nalidixic acid and 100 µg/ml for streptomycin. To estimate fitness costs competitions were performed during 24 hours in 50 ml screw-cap tubes containing 10 ml of LB medium at 37°C, with aeration (orbital shaker at 230 RPM). To estimate the frequency of each strain, in the beginning and by the end of the competition, Tetrazolium Agar (TA) medium containing 1% peptone, 0.1% yeast extract, 0.5% sodium chloride, 1.7% agar, 1% arabinose and 0.005% Tetrazolium chloride was used. All sets of dilutions were done in MgSO_4_ at a concentration of 0.01 M.

### Isolation of P1 transducted and spontaneous resistant clones

The measure of two-locus epistasis requires the construction of double mutants from single mutants, in order to obtain clones with the same mutations alone and in combination. The antibiotics chosen to isolate the mutants were rifampicin, nalidixic acid and streptomycin. Sets of 40 single clones resistant to each antibiotic were obtained by growing independent cultures of *Escherichia coli* K12 MG1655, plating in Luria-Bertani (LB) agar medium supplemented with each antibiotic and randomly selecting the clones after 24 hours of incubation at 37°C. Each clone was streak plated, and a single colony was grown in a screw-cap tube with 10 ml of LB medium supplemented with the respective antibiotic and stored in 15% glycerol at −80°C. Generalized transduction of the resistance mutations with bacteriophage P1 was performed as described previously [40]. These mutations were obtained by isolating spontaneous resistant clones and then using these as donor or recipient strains for the construction of the double mutants. Whereas in the great majority of clones the transduction efficiency was high, in 5 combinations of clones it was extremely low. These clones were termed synthetic sub-lethals and are indicated on Figure 2C. For two combinations of double resistance mutations, three independent clones were assayed for fitness. No significant differences were observed (Kruskal-Wallis test) between these clones.

Three single spontaneous clones, resistant to each antibiotic, were used to put back the resistance on the wild-type sensitive background through P1 transduction. We then measured in 5 fold replicate competitions the fitnesses of each spontaneous mutant and the corresponding single P1 transducted clone. Pairwise comparisons, by Wilcoxon test, revealed no significant differences between the single resistance clones constructed in different ways.

We exposed the single resistant clones to a second antibiotic to select for spontaneous mutants resistant to two antibiotics. These were obtained by culturing each clone carrying resistance independently and plating in Petri-dishes with the two antibiotics. Clones with the double resistance were picked randomly. All spontaneous resistant clones were tested for a mutator phenotype and those few with evidence of high mutation rate were disregarded.

### Detection of mutations

The main target genes for resistance to rifampicin, nalidixic acid and streptomycin are *rpoB*, *gyrA* and *rpsL*, respectively. To know the mutations that confer the obtained resistances, each target was amplified and then sequenced. The primers used to amplify the portion of the *rpoB* gene encoding the main set of mutations conferring resistance to rifampicin were: 5′-CGTCGTATCCGTTCCGTTGG-3′ and 5′-TTCACCCGGATAACATCTCGTC-3′; for *gyrA* gene, which encodes for the main set of mutations conferring resistance to nalidixic acid, 5′-TACACCGGTCCACATTGAGG-3′ and 5′-TTAATGATTGCCGCCGTCGG-3′; for *rpsL* gene, that encodes for the mutations conferring streptomycin resistance, 5′-ATGATGGCGGGATCGTTG-3′ and 5′-CTTCCAGTTCAGATTTACC-3′. The same primers were used for sequencing straight from the PCR product.

### Fitness assays

To measure fitness cost of the resistance mutations a competition assay was done. The resistant mutants were competed against a reference strain, *Escherichia coli* K12 MG1655 Δara in an antibiotic free environment, in an approximate proportion of 1∶1. To do so, we grew both resistant and reference strains in LB liquid medium for 24 hours at 37°C with aeration. Accurate values of each strain initial ratio were estimated by plating a dilution of the mixture in TA Agar plates. Competitions were performed in 50 ml screw-cap tubes containing 10 ml of LB liquid medium by a period of 24 hours at 37°C with aeration. By the end of this competition process, appropriate dilutions were platted onto TA agar plates to obtain the final ratios of resistants and reference strains. The fitness cost of each mutant strain- i.e the selection coefficient - was estimated as the per generation difference in Malthusian parameters for the resistant strain and the marker strain [12], discounted by the cost of the Δara marker. The fitness cost was estimated as an average of four and five independent competition assays for P1 and spontaneous resistant clones respectively. No correlation was observed between the cost of the resistance mutations and the frequency at which they arose (Figure S1).

### Measure of epistasis and statistical significance

Pairwise epistasis, *ε*, can be measured assuming a multiplicative model in which case: *ε* = *W_AB_W_ab_*−*W_Ab_W_aB_*, where *W_ij_* is the fitness of the clone carrying alleles *i* and *j* and capital letters represent the wild-type sensitive alleles. Error (σ_ε_) of the value of *ε* is then estimated by the method of error propagation:
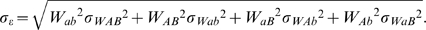
Whenever the value of *ε* was within the error we considered that alleles *a* and *b* did not show any significant epistasis (we indicate such combinations as white boxes labeled no epistasis in Figure 2C).

From the distribution of values of *ε*, provided in Figure 2B, we calculated the median value of *ε* and its 95% CI by bootstrap where we took 1000 samples. We tested normality of the distribution by a Shapiro-Wilk normality test (P = 0.024), and a Wilcoxon test for the location in zero resulted in a P value of P = 0.0006.

To query about the presence of sign epistasis in the data we made pairwise comparisons between the fitness of each double resistance clone and its corresponding single resistance clones using a Wilcoxon test to assess if the fitness of the double resistant was higher than the fitness of any of the single resistance clones. The P values are indicated in Figure 3 for those combinations that provided significant results, at 5% confidence level, after Bonferroni correction (n = 2 comparisons). For some of the double resistant clones created by P1 transduction that indicated the presence of sign epistasis- see Figure 3- we also obtained spontaneous double resistant mutants, that equally indicated evidence for sign epistasis.

Epistasis is sometimes calculated assuming an additive model [41]: *ε* = *c_ab_*−(*c_a_*+*c_b_*), such that it measures the deviation of the cost of carrying double resistance from the sum of the costs of each resistance. Since the values of the majority of the cost are small, applying the multiplicative or the additive model leads to the same conclusions, i.e. those combinations of alleles that lead to positive (negative) epistasis under the multiplicative model, also lead to positive (negative) epistasis under the additive model. A Kolmogorov-Smirnov comparing the distributions of *ε* under multiplicative model with *ε* under the additive model results on P = 0.9. The median value in the distribution of epistasis calculated under the additive model is 0.025 with bootstrap 95% CI [0.015; 0.036] and a Wilcoxon test for location strongly supports the presence of positive epistasis: P = 0.00002. This shows that the same pattern occurs applying either the multiplicative or the additive models.

To perform the statistical analysis we used the free software R: http://www.r-project.org.

## Supporting Information

Figure S1 Frequency of appearance of spontaneous mutations as a function of their fitness costs.
(0.04 MB DOC)

Figure S2 Comparison between double resistant spontaneous clones and the corresponding double mutants constructed by P1 transduction.
(0.08 MB DOC)

Figure S3 Evidence of positive epistasis in spontaneous double resistant clones.
(0.05 MB DOC)

Table S1 Genotypes and costs of single resistant mutations.
(0.05 MB DOC)

Table S2 Results of χ^2^ test on the effects of genetic background (wild type versus antibiotic resistant) on the spectrum of mutations that spontaneously arise.
(0.03 MB DOC)

## References

[1] Jasnos L, Korona R (2007). Epistatic buffering of fitness loss in yeast double deletion strains.. Nat Genet.

[2] St Onge RP, Mani R, Oh J, Proctor M, Fung E (2007). Systematic pathway analysis using high-resolution fitness profiling of combinatorial gene deletions.. Nat Genet.

[3] Zeyl C (2007). How missing genes interact.. Nature Genetics.

[4] Gagneux S, Long CD, Small PM, Van T, Schoolnik GK (2006). The competitive cost of antibiotic resistance in Mycobacterium tuberculosis.. Science.

[5] Yeh P, Tschumi AI, Kishony R (2006). Functional classification of drugs by properties of their pairwise interactions.. Nat Genet.

[6] Chait R, Craney A, Kishony R (2007). Antibiotic interactions that select against resistance.. Nature.

[7] Williams RJ, Heymann DL (1998). Containment of antibiotic resistance.. Science.

[8] Andersson DI, Levin BR (1999). The biological cost of antibiotic resistance.. Curr Opin Microbiol.

[9] Bonhoeffer S, Lipsitch M, Levin BR (1997). Evaluating treatment protocols to prevent antibiotic resistance.. Proc Natl Acad Sci U S A.

[10] Kurland CG, Hughes D, Ehrenberg M (1996). Limitations of translational accuracy.

[11] Siddiqi N, Shamim M, Hussain S, Choudhary RK, Ahmed N (2002). Molecular characterization of multidrug-resistant isolates of Mycobacterium tuberculosis from patients in North India.. Antimicrob Agents Chemother.

[12] Lenski RE, Rose MR, Simpson SC, Tadler SC (1991). Long-Term Experimental Evolution In Escherichia-Coli.1. Adaptation And Divergence During 2,000 Generations.. American Naturalist.

[13] Eyre-Walker A, Keightley PD (2007). The distribution of fitness effects of new mutations.. Nat Rev Genet.

[14] Maisnier-Patin S, Berg OG, Liljas L, Andersson DI (2002). Compensatory adaptation to the deleterious effect of antibiotic resistance in Salmonella typhimurium.. Mol Microbiol.

[15] Reynolds MG (2000). Compensatory evolution in rifampin-resistant Escherichia coli.. Genetics.

[16] Levin BR, Perrot V, Walker N (2000). Compensatory mutations, antibiotic resistance and the population genetics of adaptive evolution in bacteria.. Genetics.

[17] Kouyos RD, Silander OK, Bonhoeffer S (2007). Epistasis between deleterious mutations and the evolution of recombination.. Trends Ecol Evol.

[18] Wilke CO, Adami C (2001). Interaction between directional epistasis and average mutational effects.. Proc Biol Sci.

[19] Weinreich DM, Watson RA, Chao L (2005). Perspective: Sign epistasis and genetic constraint on evolutionary trajectories.. Evolution.

[20] Weinreich DM, Delaney NF, Depristo MA, Hartl DL (2006). Darwinian evolution can follow only very few mutational paths to fitter proteins.. Science.

[21] Bjorkman J, Nagaev I, Berg OG, Hughes D, Andersson DI (2000). Effects of environment on compensatory mutations to ameliorate costs of antibiotic resistance.. Science.

[22] Schrag SJ, Perrot V (1996). Reducing antibiotic resistance.. Nature.

[23] MacLean R, Buckling A (2009). The distribution of fitness effects of beneficial mutations in Pseudomonas aeruginosa.. PLoS Genet.

[24] Crow J, Kimura M (1970). An Introduction to Population Genetics Theory.

[25] Perfeito L, Fernandes L, Mota C, Gordo I (2007). Adaptive mutations in bacteria: high rate and small effects.. Science.

[26] Sekiguchi J, Miyoshi-Akiyama T, Augustynowicz-Kopec E, Zwolska Z, Kirikae F (2007). Detection of multidrug resistance in Mycobacterium tuberculosis.. J Clin Microbiol.

[27] Dominguez E, Zarazaga M, Saenz Y, Brinas L, Torres C (2002). Mechanisms of antibiotic resistance in Escherichia coli isolates obtained from healthy children in Spain.. Microb Drug Resist.

[28] Hopkins KL, Arnold C, Threlfall EJ (2007). Rapid detection of gyrA and parC mutations in quinolone-resistant Salmonella enterica using Pyrosequencing technology.. J Microbiol Methods.

[29] Kondrashov AS (1988). Deleterious mutations and the evolution of sexual reproduction.. Nature.

[30] Elena SF, Lenski RE (1997). Test of synergistic interactions among deleterious mutations in bacteria.. Nature.

[31] Segre D, Deluna A, Church GM, Kishony R (2005). Modular epistasis in yeast metabolism.. Nat Genet.

[32] Bonhoeffer S, Chappey C, Parkin NT, Whitcomb JM, Petropoulos CJ (2004). Evidence for positive epistasis in HIV-1.. Science.

[33] Ward H, Perron GG, Maclean RC (2009). The cost of multiple drug resistance in Pseudomonas aeruginosa.. Journal of Evolutionary Biology.

[34] Rozen DE, McGee L, Levin BR, Klugman KP (2007). Fitness costs of fluoroquinolone resistance in Streptococcus pneumoniae.. Antimicrobial Agents and Chemotherapy.

[35] Chakrabarti SL, Gorini L (1977). Interaction between mutations of ribosomes and RNA polymerase: a pair of strA and rif mutants individually temperature-insensitive but temperature-sensitive in combination.. Proc Natl Acad Sci U S A.

[36] Kumaresan KR, Jayaraman R (1988). SOS independent survival against mitomycin C induced lethality in a rifampicin-nalidixic acid-resistant mutant of Escherichia coli.. Mutat Res.

[37] Schrag SJ, Perrot V, Levin BR (1997). Adaptation to the fitness costs of antibiotic resistance in Escherichia coli.. Proceedings of the Royal Society of London Series B-Biological Sciences.

[38] Hegreness M, Shoresh N, Damian D, Hartl D, Kishony R (2008). Accelerated evolution of resistance in multidrug environments.. Proceedings of the National Academy of Sciences of the United States of America.

[39] Michel JB, Yeh PJ, Chait R, Moellering RC, Kishony R (2008). Drug interactions modulate the potential for evolution of resistance.. Proceedings of the National Academy of Sciences of the United States of America.

[40] Silhavy T, Berman M, Enquist L (1984). Experiments with gene fusions. Experiments with gene fusion.

[41] Phillips PC (2008). Epistasis - the essential role of gene interactions in the structure and evolution of genetic systems.. Nature Reviews Genetics.

