# Supplementary material for: Positive Epistasis Drives the Acquisition of Multidrug Resistance
Source: PLoS Genet. 2009 Jul 24;5(7):e1000578. doi: 10.1371/journal.pgen.1000578 (PMC2706973; doi:10.1371/journal.pgen.1000578)
Supplement: Figure S1 — Frequency of appearance of spontaneous mutations as a function of their fitness costs. (0.04 MB DOC) [file pgen.1000578.s001.doc]

**Figure S1.**

**
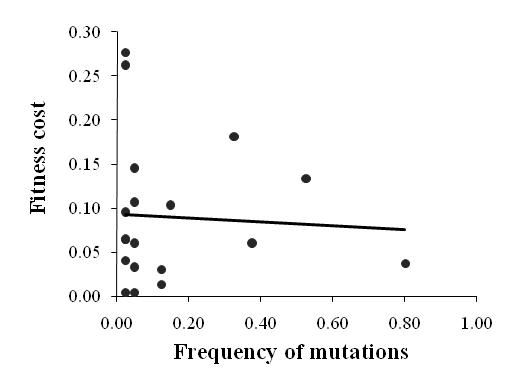
**

Figure S1. Frequency of appearance of spontaneous mutations as a function of their fitness costs. Linear regression: slope -0.02 + 0.09(SE).
