# Supplementary material for: Positive Epistasis Drives the Acquisition of Multidrug Resistance
Source: PLoS Genet. 2009 Jul 24;5(7):e1000578. doi: 10.1371/journal.pgen.1000578 (PMC2706973; doi:10.1371/journal.pgen.1000578)
Supplement: Figure S2 — Comparison between double resistant spontaneous clones and the corresponding double mutants constructed by P1 transduction. (0.08 MB DOC) [file pgen.1000578.s002.doc]

**Figure S2.**

Figure S2. Comparison between double resistant spontaneous clones and the corresponding double mutants constructed by P1 transduction. Wilcoxon signed rank tests were made for assaying fitness differences between every pair of mutants constructed by the two independent methods. At a significance level of α=0.01, 5 spontaneous resistance clones were found to have higher fitness than the corresponding P1 transducted clones, and are therefore candidates to carry compensatory mutations.
