# Supplementary material for: Positive Epistasis Drives the Acquisition of Multidrug Resistance
Source: PLoS Genet. 2009 Jul 24;5(7):e1000578. doi: 10.1371/journal.pgen.1000578 (PMC2706973; doi:10.1371/journal.pgen.1000578)
Supplement: Figure S3 — Evidence of positive epistasis in spontaneous double resistant clones. (0.05 MB DOC) [file pgen.1000578.s003.doc]

**Figure S3.**


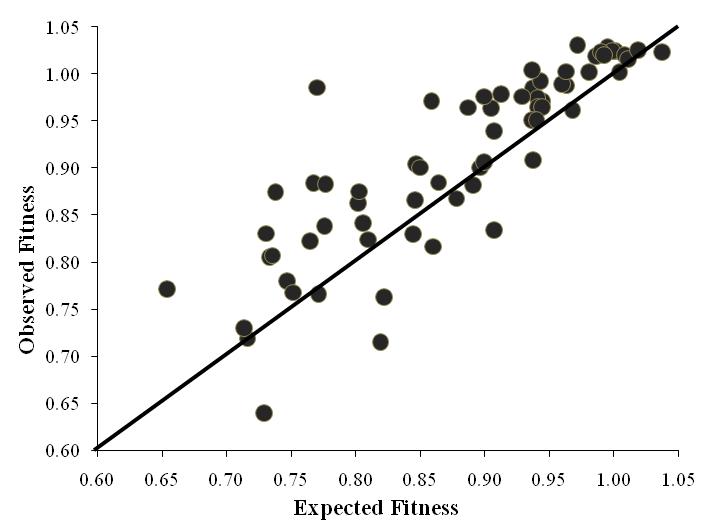


Figure S3. Relation between the observed fitness of the 67 spontaneous double resistance genotypes and the expected fitness under the assumption of no epistasis (compensated and synthetic sub-lethals clones are excluded from the data).
