# Supplementary material for: Positive Epistasis Drives the Acquisition of Multidrug Resistance
Source: PLoS Genet. 2009 Jul 24;5(7):e1000578. doi: 10.1371/journal.pgen.1000578 (PMC2706973; doi:10.1371/journal.pgen.1000578)
Supplement: Table S1 — Genotypes and costs of single resistant mutations. (0.05 MB DOC) [file pgen.1000578.s004.doc]

**Table S1. Genotypes and fitness costs of single resistance mutations**

| **Gene** | **Genotype**  **aa change ; nt change** | **Cost (2SE)%** | **Resistance** |
| --- | --- | --- | --- |
| *gyrA* | D 87 G ; GAC to GGC | 3.7 (1.5) | Nal |
| *gyrA* | S 83 L; TCG to TTG | 3.3 (1.2) | Nal |
| *gyrA* | D 87 Y; GAC to TAC | 3.1 (1.9) | Nal |
| *rpoB* | D 516 V; GAC to GTC | 6.0 (1.0) | Rif |
| *rpoB* | H 526 N; CAC to AAC | 1.4 (1.1) | Rif |
| *rpoB* | H 526 L; CAC to CTC | 0.4 (1.5) | Rif |
| *rpoB* | H 526 Y; CAC to TAC | 10.3 (3.2) | Rif |
| *rpoB* | I 572 F; ATC to TTC | 14.6 (1.2) | Rif |
| *rpoB* | D 516 Y; GAC to TAC | 6.4 (4.0) | Rif |
| *rpoB* | D516N; GAC to AAC | 4.1 (2.0) | Rif |
| *rpoB* | R 529 H; CGT to CAT | 26.2 (4.9) | Rif |
| *rpoB* | S 512 F; TCT to TTT | 6.5 (1.3) | Rif |
| *rpoB* | S 531 F; TCC to TTC | 10.7 (1.5) | Rif |
| *rpoB* | H 526 D; CAC to GAC | 9.6 (1.2) | Rif |
| *rpsL* | K 43 R; AAA to AGA | 0.5 (1.4) | Str |
| *rpsL* | K 43 T; AAA to ACA | 13.4 (2.0) | Str |
| *rpsL* | K 88 E; AAA to GAA | 27.5 (2.8) | Str |
| *rpsL* | K 43 N; AAA to AAC | 18.0 (1.9) | Str |
| *rpsL* | K 88 R; AAA to AGA | 6.1 (1.2) | Str |
