# Supplementary material for: Positive Epistasis Drives the Acquisition of Multidrug Resistance
Source: PLoS Genet. 2009 Jul 24;5(7):e1000578. doi: 10.1371/journal.pgen.1000578 (PMC2706973; doi:10.1371/journal.pgen.1000578)
Supplement: Table S2 — Results of χ2 test on the effects of genetic background (wild type versus antibiotic resistant) on the spectrum of mutations that spontaneously arise. (0.03 MB DOC) [file pgen.1000578.s005.doc]

**Table S2. Results of χ ^2^ test on the effects of genetic background (wild type *versus* antibiotic resistant) on the spectrum of mutations that spontaneously arise**
